# Supplementary material for: OpenMM 7: Rapid development of high performance algorithms for molecular dynamics
Source: PLoS Comput Biol. 2017 Jul 26;13(7):e1005659. doi: 10.1371/journal.pcbi.1005659 (PMC5549999; doi:10.1371/journal.pcbi.1005659)
Supplement: S1 Source Code — (ZIP) [file pcbi.1005659.s002.zip › OpenMM-7.0.1-Source/docs/api-c++/generated/OpenMM.AmoebaWcaDispersionForce.html]

AmoebaWcaDispersionForce — OpenMM @OPENMM\_MAJOR\_VERSION@.@OPENMM\_MINOR\_VERSION@ documentation


# AmoebaWcaDispersionForce¶

*class* `OpenMM::``AmoebaWcaDispersionForce`¶
:   This class implements a nonbonded interaction between pairs of particles typically used along with `AmoebaGeneralizedKirkwoodForce` as part of an implicit solvent model.

    To use it, create an `AmoebaWcaDispersionForce` object then call `addParticle()` once for each particle. After a particle has been added, you can modify its force field parameters by calling `setParticleParameters()`. This will have no effect on Contexts that already exist unless you call `updateParametersInContext()`.

    Methods

    |  |  |
    | --- | --- |
    | `AmoebaWcaDispersionForce` | Create an `AmoebaWcaDispersionForce`. |
    | `getNumParticles` | Get the number of particles |
    | `setParticleParameters` | Set the force field parameters for a WCA dispersion particle. |
    | `getParticleParameters` | Get the force field parameters for a WCA dispersion particle. |
    | `addParticle` | Set the force field parameters for a WCA dispersion particle. |
    | `updateParametersInContext` | Update the per-particle parameters in a `Context` to match those stored in this `Force` object. |
    | `getEpso` |  |
    | `getEpsh` |  |
    | `getRmino` |  |
    | `getRminh` |  |
    | `getAwater` |  |
    | `getShctd` |  |
    | `getDispoff` |  |
    | `getSlevy` |  |
    | `setEpso` |  |
    | `setEpsh` |  |
    | `setRmino` |  |
    | `setRminh` |  |
    | `setAwater` |  |
    | `setShctd` |  |
    | `setDispoff` |  |
    | `setSlevy` |  |
    | `usesPeriodicBoundaryConditions` | Returns whether or not this force makes use of periodic boundary conditions. |

    `AmoebaWcaDispersionForce`()¶
    :   Create an `AmoebaWcaDispersionForce`.

    int `getNumParticles`() *const*¶
    :   Get the number of particles

    void `setParticleParameters`(int *particleIndex*, double *radius*, double *epsilon*)¶
    :   Set the force field parameters for a WCA dispersion particle.

        |  |  |
        | --- | --- |
        | Parameters: |  |

        - **particleIndex** – the particle index
        - **radius** – radius
        - **epsilon** – epsilon

    void `getParticleParameters`(int *particleIndex*, double &*radius*, double &*epsilon*) *const*¶
    :   Get the force field parameters for a WCA dispersion particle.

        |  |  |
        | --- | --- |
        | Parameters: |  |

        - **particleIndex** – the particle index
        - **radius** – [out] radius
        - **epsilon** – [out] epsilon

    int `addParticle`(double *radius*, double *epsilon*)¶
    :   Set the force field parameters for a WCA dispersion particle.

        |  |  |
        | --- | --- |
        | Parameters: |  |

        - **radius** – radius
        - **epsilon** – epsilon

        |  |  |
        | --- | --- |
        | Returns: | index of added particle |

    void `updateParametersInContext`(Context &*context*)¶
    :   Update the per-particle parameters in a `Context` to match those stored in this `Force` object. This method provides an efficient method to update certain parameters in an existing `Context` without needing to reinitialize it. Simply call `setParticleParameters()` to modify this object’s parameters, then call `updateParametersInContext()` to copy them over to the `Context`.

        The only information this method updates is the values of per-particle parameters. All other aspects of the `Force` are unaffected and can only be changed by reinitializing the `Context`.

    double `getEpso`() *const*¶

    double `getEpsh`() *const*¶

    double `getRmino`() *const*¶

    double `getRminh`() *const*¶

    double `getAwater`() *const*¶

    double `getShctd`() *const*¶

    double `getDispoff`() *const*¶

    double `getSlevy`() *const*¶

    void `setEpso`(double *inputValue*)¶

    void `setEpsh`(double *inputValue*)¶

    void `setRmino`(double *inputValue*)¶

    void `setRminh`(double *inputValue*)¶

    void `setAwater`(double *inputValue*)¶

    void `setShctd`(double *inputValue*)¶

    void `setDispoff`(double *inputValue*)¶

    void `setSlevy`(double *inputValue*)¶

    bool `usesPeriodicBoundaryConditions`() *const*¶
    :   Returns whether or not this force makes use of periodic boundary conditions.

        |  |  |
        | --- | --- |
        | Returns: | true if nonbondedMethod uses PBC and false otherwise |

High performance molecular simulation on GPUs


### Navigation

- Core Objects
- Forces
- Integrators
- Extras

©2015, Stanford University and the Authors.
|
Powered by Sphinx 1.3.1
& Alabaster 0.7.7
|
Page source
